# Interactive Effect of C-Reactive Protein upon the Relationship Between *Chlamydia trachomatis* and Depression

**DOI:** 10.3390/diagnostics15202638

**Published:** 2025-10-19

**Authors:** Kay Banerjee, W. Sumner Davis, Sri Banerjee

**Affiliations:** 1 School of Counseling, Walden University, Minneapolis, MN 55401, USA; karen.banerjee@waldenu.edu; 2 School of Health Sciences and Public Health, Walden University, Minneapolis, MN 55401, USA; dr.sumner.davis@gmail.com

**Keywords:** inflammation, sexually transmitted infections, depression, mental health, NHANES

## Abstract

**Introduction**: *Chlamydia trachomatis* is an important indicator of overall health and plays a vital role in various health conditions. In 2018, *C. trachomatis* reached the highest level ever recorded, resulting in USD 691 million in expenditures, with 1.8 million reported cases. This amount reflects a 19% increase since 2014, according to the Centers for Disease Control and Prevention. Depression has also been on the rise between 2013 (8.2%) to 2023 (13.1%). *C. trachomatis* and depression may have inflammation as a final common pathway. The purpose of this study was to explore a potential connection between *C. trachomatis* and depression, and whether C-reactive protein (CRP) modifies this effect. **Methods**: For this study, we utilized the 2005–2010 National Health and Nutrition Examination Survey (NHANES) and US adults aged between 20 and 59 years. Depression was determined from the Patient Health Questionnaire-9 (PHQ-9). Complex Samples logistic regression models were used to determine if *C. trachomatis* in the previous year was predictive of depression. Models were adjusted for known confounders, including age, gender, race/ethnicity, poverty, and education. **Results**: For the presence of depression, the unadjusted Odds Ratio (OR) for *C. trachomatis* to no *C. trachomatis* was OR = 2.9. The adjusted OR was elevated, at OR = 6.3 among individuals who had both *C. trachomatis* and elevated CRP, but close to 1.0 among individuals who had *C. trachomatis* but reported low CRP after adjusting for demographic and health variables. **Conclusions**: Using a nationally representative sample, our study was the first to demonstrate, across unadjusted and adjusted models, that there is a strong connection between a history of *C. trachomatis* and high CRP leading to worse outcomes in individuals with depression than *C. trachomatis* infection alone. This finding indicates the need to conduct mental health screening among individuals with Sexually Transmitted Infections (STIs) or other infectious diseases.

## 1. Introduction

Sexually Transmitted Infections (STIs) have been increasing in the United States [1,2]. According to Chang et al. (2020), *C. trachomatis* is one of the most diagnosed STIs in the US and worldwide [3]. Additionally, depression is also on the rise [1]. Consequently, Anderer (2025) notes that depression has grown by about sixty percent in recent years [4]. The interaction between infectious diseases, inflammation, and mental health disorders has become a growing area of research [5,6,7]. Miller et al. (2023) found that the therapeutic potential of targeting the immune system to treat depression can aid in reinventing psychiatric drug therapy strategies [8]. This connection demonstrates the need to further assess how addressing inflammation can lead to more robust and complete interventions to treat both *C. trachomatis* and depression. *C. trachomatis* infections are known to trigger chronic inflammatory responses, which may contribute to mental health issues, including depression [7]. Steffens et al. wrote a review article demonstrating a strong connection between mental health and *C. trachomatis* [9]. Understanding how *C. trachomatis* infections and the resulting inflammatory processes are linked to depression is essential for developing comprehensive treatment strategies. In an original study, authors found that across a study cohort of 839 individuals, those with *C. trachomatis* had a 1.79 higher likelihood of depression than individuals without *C. trachomatis* [10,11]. Ghosh et al. (2025) also found this to be true among vulnerable pregnant populations [11]. Concurrently, Ling et al. (2022) found that there was an association between *C. trachomatis* and inflammation, potentially leading to tubal infertility [12]. Rodrigues et al. (2022) synthesized the current peer-reviewed literature to find the biological mechanisms and inflammatory correlations potentially triggering infertility, as well as gynecological tumors [13].

### Inflammatory Mechanisms Induced by C. trachomatis

*C. trachomatis* is an intracellular bacterial pathogen that triggers a persistent immune response in the host. Studies have shown that *C. trachomatis* infections activate inflammasomes, leading to the secretion of pro-inflammatory cytokines, such as interleukin-1 beta (IL-1β) and interleukin-18 (IL-18) [14,15]. These cytokines are central to the body’s immune response and contribute to systemic inflammation. Chronic *C. trachomatis* infections, particularly when untreated, can lead to conditions such as pelvic inflammatory disease (PID), which is associated with prolonged inflammatory states [16,17].

There is substantial evidence that chronic inflammation plays a role in the pathophysiology of depression [18,19]. There is also evidence that there may be a direct link between *C. trachomatis* and depression due to the potential of *C. trachomatis* to cross the blood–brain barrier, directly affecting the brain and mood disorders. However, what is not known is how *C. trachomatis* interacting with inflammation may directly lead to depression. Therefore, the purpose of this study was to use a nationally representative dataset to evaluate the relationship between *C. trachomatis* infection (measured by a history of *C. trachomatis*) and depression, and its connection to inflammation. To our knowledge, no population-level studies have examined this relationship. We hypothesize that there will be an association between *C. trachomatis* and depression, and CRP will modify this effect. Understanding this connection may guide future interventions to improve healthcare sites where individuals seek STI screening and treatment.

## 2. Methods

The study sample is representative of adults aged 20 to 59 years, since this is the age range during which *C. trachomatis* occurs. Additionally, this follows the Strengthening the Reporting of Observational Studies in Epidemiology (STROBE) guidelines. Before the data collection, the National Health and Nutrition Examination Survey (NHANES) procedures were approved by the National Center for Health Statistics (NCHS). The publicly available data was obtained through the Centers for Disease Control and Prevention (CDC) website. Additionally, ethical approval for the data analysis was granted by the Walden University Institutional Review Board (protocol code: 03-01-22-2263753).

Depression was the primary outcome variable, ascertained using the Patient Health Questionnaire-9 (PHQ-9) from responses to the NHANES questionnaire [20]. It was created and developed by Drs. Robert L. Spitzer, Janet B.W. Williams, and Kurt Kroenke in 1999 [20] and was funded by a grant from Pfizer. It is a 9-item, self-report questionnaire adapted from the longer PHQ assessment, which itself was a self-administered version of the PRIME-MD diagnostic tool. The PHQ-9 was designed to be a brief, practical tool for identifying and monitoring depression severity in primary care settings. This was initially as a screening question “has a doctor or other health professional ever told you that you have [disease]?”. Each question asks about a specific symptom of depression over the past two weeks, with responses ranging from “not at all” to “nearly every day”. *C. trachomatis* was determined by the question “In the past 12 months, has a doctor or other healthcare professional told you that you had *C. trachomatis*?”

Detection of CRP was performed by latex-enhanced nephelometry corresponding to the continuous NHANES data year, which was part of the publicly available dataset. This was collected using a Siemens/Behring Nephelometer, Munich, Germany, which allowed a conversion of the intensity of reflected light to milligrams of CRP per deciliter (mg/dL). The lowest reportable value was 0.02 mg/dL; everyone at or below the detection limit had a value reported as 0.01 mg/dL. Results were converted to mg/L to provide consistency with American Heart Association (AHA) risk categories. Equipment or reporting changes were not made during the three cycles of data used. Inflammatory biomarker CRP was determined at a previously validated cutoff point of ≥2 mg/dL (for elevated levels).

Similarly, covariates, such as cardiovascular disease history, were determined by the self-reported diagnosis of coronary heart disease, angina, stroke, congestive heart failure, or myocardial infarction. For chronic kidney disease (CKD), the glomerular filtration rate was derived from the CKD-EPI equation using measured creatinine levels. Cardiovascular disease (CVD) was one of the confounders and was identified using the Medical Conditions Questionnaire (MCQ), which includes self-reported diagnoses of the following: congestive heart failure—“Has a doctor or other health professional ever diagnosed you with congestive heart failure?”; coronary heart disease—“Has a doctor or other health professional ever diagnosed you with coronary heart disease?”; angina (angina pectoris)—“Has a doctor or other health professional ever diagnosed you with angina?”; and heart attack (myocardial infarction)—“Has a doctor or other health professional ever diagnosed you with a heart attack?”

Major health-related and demographic variables were also included in this analysis. Data on body mass index (BMI) was derived from measured height and weight and categorized into four groups (i.e., BMI < 25 kg/m^2^ = normal weight; BMI = 25–29 kg/m^2^ = overweight; BMI = 30–39.9 kg/m^2^ = obese; and participants with BMI > 40 kg/m^2^ were considered severely obese). For the multivariate models, obesity was dichotomized and considered present for BMI ≥ 30 kg/m^2^ and considered absent for the rest. A two-variable indicator of current smoking status was created with “nonsmoker” (coded 0), “former smoker” (coded 1), and “nonsmoker (2). The subject was considered a “smoker” if s/he reported “yes” to the question, “Have you smoked at least 100 cigarettes in your entire life?” and answered, “every day” and “some days” to the question, “Do you now smoke cigarettes…”.

For demographic variables to be considered as covariates, age (continuous), sex (categorical), education (categorical), income (categorical), and ethnicity (categorical) were utilized. Ethnicity was divided into “Non-Hispanic White”, “Non-Hispanic Black”, “Hispanic”, “Asian”, and others. Education-level data was categorized into three groups: “Less Than High School” versus “High School Graduate” versus “Some College or Above”. Income information was computed by the poverty income ratio (PIR), which is also an indicator of income relative to the economic needs of a household. This is a polytomous variable that was achieved by calculating annual fluctuations in household size and cost of living and monitoring the consumer price index concerning household income and federally established poverty limitations. PIR levels were dichotomized for analysis with a cutoff point of 1.

### Statistical Analysis

Weighted demographic variables were applied to approximate distributions in the USA by using the provided sample weights (to account for oversampling of certain groups, unequal probabilities of selection, and non-response). Normal distributions of values were assessed by using the Shapiro–Wilk test. Categorical variables were expressed as percentage values and analyzed using chi-square tests. Complex samples logistic regression models were used to examine the risk of depression based on *C. trachomatis* status or inflammation after adjusting for covariates. Statistical analyses were conducted using the SAS System for Windows (release 9.3; SAS Institute Inc., Cary, NC, USA) and SUDAAN (release 9.0; Research Triangle Institute, Research Triangle Park, NC, USA). Additionally, we generated bar graphs. Statistical significance for tests was considered at a *p*-level of <0.05.

## 3. Results

**Figure 1:**
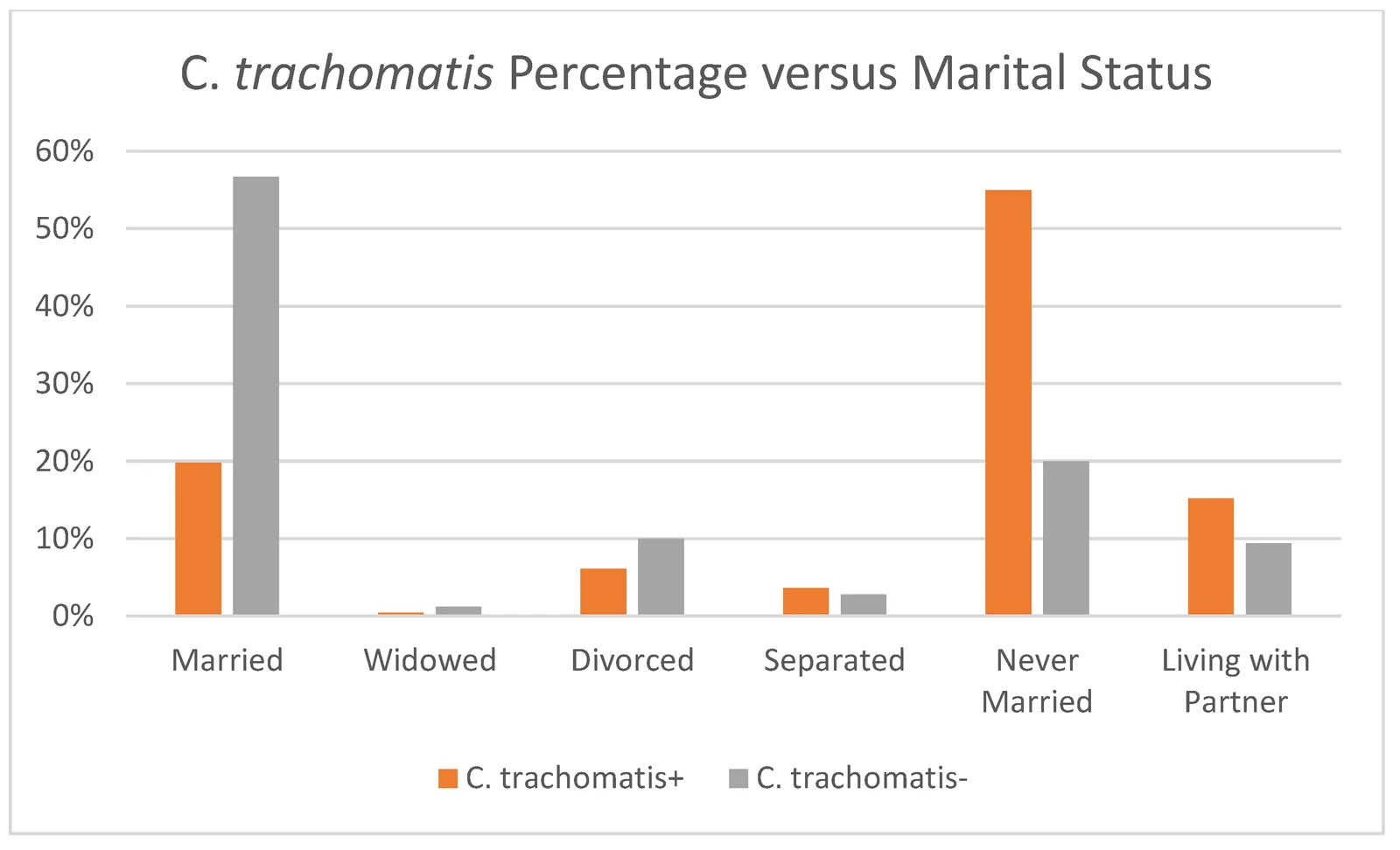
*C. trachomatis* status versus marital status.

After combining data from the NHANES 2005–2010, we had an unweighted sample size of 25,938 individuals aged 20 to 59 years with complete medical examination data. As seen in Table 1, less than a tenth (10%) had a positive *C. trachomatis* status, and 12.4% had high CRP. Table 1 displays the demographic characteristics of the sample. Approximately 50% were women, 67.5% were non-Hispanic white, and their mean age was 30.2 years. According to Figure 1, there were differences between the percentage with *C. trachomatis* and marital status (among people who were married, individuals with *C. trachomatis* were much lower (19.8%) vs. married w/o *C. trachomatis* (56.7%)). Also, when dichotomized, people with *C. trachomatis* were more likely to have CVD, diabetes, and depression. Also, as shown in Table 1, among those with positive *C. trachomatis*, a higher proportion had depression (15.3% vs. 7.9%, *p* < 0.01) than those without depression. As established in Figure 1, as an incidental finding we determined that married (20%) individuals had a significantly lower percentage of *C. trachomatis* than individuals that were never married (55%).

**Figure 2:**
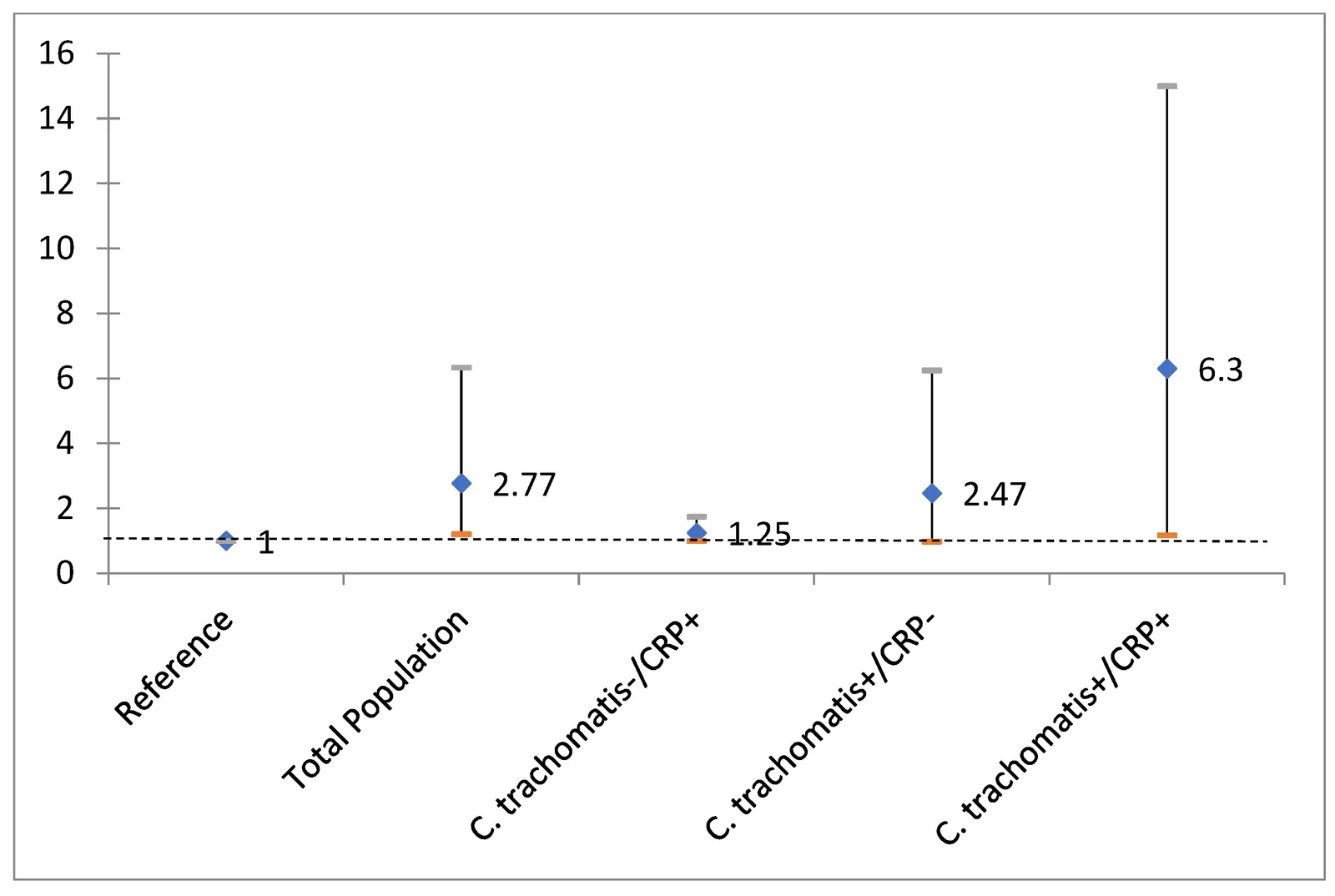
Crude Odds Ratio (95% CI) between different levels of CRP and *C. trachomatis* status. For better visualization, the (95% CI) upper limit was rounded down to 15 from 33.5. Blue dot represents the corresponding Odds Ratios while the bars represent CI.

As seen in Figure 2 and Table 2, for depression, the unadjusted odds ratio (OR) for *C. trachomatis* to no *C. trachomatis* was 2.86 (95% confidence interval [CI], 1.55–5.31, *p* < 0.001). Even after adjustment, the OR for *C. trachomatis* to no *C. trachomatis* remained strong (OR = 2.73). As seen in Table 2 and Figure 2, the adjusted OR was elevated 6.30 (CI 1.18–33.49, *p* < 0.05) among individuals who had *C. trachomatis* and a high CRP, but close to 1.0 (1.25 CI 1.00–1.75, *p* = 0.59) among individuals who had a history of *C. trachomatis* but reported low CRP after adjusting for demographic (smoking status, chronic kidney disease, CVD, diabetes, BMI, poverty–income ratio, age, marital status, ethnicity education status, and gender) and health variables (smoking status, chronic kidney disease, CVD, and diabetes).

## 4. Discussion

To our knowledge, this was the first nationally representative study that found that *C. trachomatis* is connected to depression. We also found that there is substantial evidence that chronic inflammation plays an interactive role in the pathophysiology of STIs such as *C. trachomatis* and depression. CRP is an important indicator of generalized inflammation and mental health, as has been shown in previous studies [9,10,11]. Similarly, the cytokine hypothesis suggests that pro-inflammatory markers, such as IL-6, tumor necrosis factor-alpha (TNF-α), and CRP [21], contribute to alterations in neurotransmitter systems and neural plasticity, which may lead to depressive symptoms [15]. Elevated levels of these cytokines have been found in individuals diagnosed with major depressive disorder (MDD), further supporting the link between inflammation and depression [22].

### 4.1. Neuroimmune Interactions in Depression

Also, our study was the first to demonstrate that there is a statistically significant association between *C. trachomatis and* depression. Additionally, we found that high levels of CRP have worse outcomes than *C. trachomatis* alone. We also found that those individuals who were single were more likely to have *C. trachomatis* than those who were married. This finding indicates the need to conduct mental health screening among individuals with STIs or other conditions. Inflammation can influence the central nervous system (CNS) by altering the metabolism of tryptophan, a precursor to serotonin. Pro-inflammatory cytokines induce the kynurenine pathway, leading to an increased production of neurotoxic metabolites such as quinolinic acid while reducing serotonin availability [8,17]. This mechanism has been proposed as a potential explanation for the increased prevalence of depression in individuals with chronic inflammatory diseases, including those caused by *C. trachomatis* infections.

### 4.2. Epidemiological Evidence Linking C. trachomatis, Inflammation, and Depression

Recent epidemiological studies support the association between *C. trachomatis* infections, inflammatory disorders, and depression. Darville (2021) [23] found that individuals with severe depression had over six times the odds of having Pelvic Inflammatory Disease (PID) compared to those without depression. This study highlights a significant association between *C. trachomatis*-induced inflammatory conditions and mental health outcomes. Similarly, research has shown that women diagnosed with sexually transmitted infections (STIs) report higher rates of depressive symptoms compared to those without STIs, reinforcing the psychological burden of chronic infections [22,24].

### 4.3. Recommendations and Practical Implications

Given the connection between *C. trachomatis*, inflammation, and depression, integrated treatment approaches may be beneficial. Traditional antibiotic therapy for *C. trachomatis* (e.g., doxycycline or azithromycin) can effectively resolve the infection, but addressing inflammation-related depressive symptoms may require adjunctive anti-inflammatory or psychotropic treatments [25]. Clinical trials examining the efficacy of anti-inflammatory medications, such as nonsteroidal anti-inflammatory drugs (NSAIDs) and cytokine inhibitors, have shown promise in alleviating depressive symptoms in patients with high inflammatory markers [24,25]. This suggests that targeting inflammation in patients with *C. trachomatis*-related depression could be a viable therapeutic strategy. In addition, as was established in our incidental findings, married individuals were less likely to have *C. trachomatis* than individuals who were never married. It could be hypothesized that unmarried people tend to feel lonelier or have attachment patterns that prevent them from being in stable relationships, and that these factors may contribute to greater stress or depression. Also, it is important to promote condom usage and preventative measures for individuals who are not married. Also, there should be more screening for *C. trachomatis* by primary care practitioners. In addition, there should be a higher index of suspicion among individuals in the age group of 20- to 24-year-olds. Since females have a higher reported rate of *C. trachomatis* than males due to increased symptoms, males are not regularly screened. However, screening should be improved among males and females. Currently, the U.S. Preventive Services Task Force (USPSTF) recommends annual *C. trachomatis* screening for all sexually active women 24 years and younger, since *C. trachomatis* can lead to serious inflammatory complications. For women aged 25 and older, screening is recommended if risk factors are present, such as new or multiple sex partners. However, for men, targeted screening is considered, such as for sexually active young men in high-prevalence clinics and for men who have sex with men. Therefore, we recommend that annual *C. trachomatis* screening be performed for males, as well, especially since *C. trachomatis* can be spread asymptomatically. There is also potential for *C. trachomatis* and gonorrhea coinfection, requiring differential treatment. This is common, especially for people with gonorrhea, and requires co-treatment with a single dose of ceftriaxone for gonorrhea and a seven-day course of oral doxycycline for *C. trachomatis*.

### 4.4. Potential Limitations

This study has some limitations. First, the NHANES is self-reported, which may reflect recall bias [26]. In addition, there is a potential for social desirability bias, as many individuals may not want to report the fact that they had *C. trachomatis*, and bias in self-reported data [27]. Another limitation is that correlation does not mean causation. Further studies may include an experimental study design that directly shows causation. Also, healthcare practitioners should screen for mental health during STI treatment and also look for inflammatory measures such as CRP [10,28]. Patient–physician communication is especially important, as partner notification should be encouraged and even required to interrupt transmission of infection. Further qualitative studies should take a look at the complexity, challenges of partner notification, and psychosocial impact of *C. trachomatis* diagnosis. Finally, the links and possible mediating or latent variables in the *C. trachomatis*–depression association require further investigation.

## 5. Conclusions

The existing body of research provides compelling evidence that *C. trachomatis* infections contribute to systemic inflammation, which in turn may elevate the risk of depression. The inflammatory pathways activated by *C. trachomatis* appear to intersect with mechanisms implicated in depressive disorders, particularly through cytokine-mediated neurotransmitter dysregulation. Epidemiological studies further confirm an association between inflammatory STIs and mental health outcomes. Moving forward, interdisciplinary research integrating infectious disease management, psychoneuroimmunology, and mental health treatment is essential for developing comprehensive care strategies.

## Figures and Tables

**Table 1:** Characteristics of study participants stratified by *C. trachomatis* status.

Characteristics	Total Population (*n* = 25,938)	*C. trachomatis* (+) (*n* = 271)	*C. trachomatis* (−) (*n* = 25,667)
**C-reactive Protein (%)**	9.2 (8.6–9.7)	12.4 (6.6–18.3)	9.1 (8.6–9.7)
**Smoking Status (%)**			
Never Smoked	53.6 (52.3–54.7)	50.3 (42.6–57.9)	53.6 (52.4–54.8)
Formerly Smoked	20.6 (19.8–21.4)	15.6 (8.4–22.7)	20.6 (19.8–21.5)
Current Smoker	25.8 (24.8–26.9)	34.1 (25.9–42.2)	25.8 (24.7–26.6)
**Chronic Kidney Disease (CKD) (%)**	7.4 (7.0–7.8)	7.8 (4.4–13.6)	7.4 (7.0–7.8)
Cardiovascular Disease (CVD) (%) *	3.7 (3.4–4.1)	1.6 (0.7–3.6)	3.8 (3.4–4.1)
Diabetes (%) **	5.4 (5.0–5.7)	1.7 (0.8–3.6)	5.4 (5.0–5.8)
**BMI (%)**			
BMI ≥ 18.5 and BMI < 25	31.7 (30.7–32.6)	36.9 (29.2–44.6)	31.6 (30.6–32.6)
BMI ≥ 25 and BMI < 29.9	33.1 (32.3–34.0)	28.9 (22.3–35.6)	33.2 (32.3–34.0)
BMI ≥ 30 and BMI < 34.9	19.6 (18.9–20.2)	17.8 (10.0–25.7)	19.6 (18.9–20.20)
BMI ≥ 35.0 and BMI < 39.9	9.0 (8.5–9.4)	8.5 (5.2–13.6)	9.0 (8.5–9.4)
BMI ≥ 40.0	6.7 (6.2–7.1)	7.8 (5.0–11.9)	6.7 (6.2–7.1)
Age (SE) **	39.6 (0.13)	30.2 (30.4)	39.6 (0.13)
**Gender Female (%)**	50.5 (49.9–51.1)	62.1 (54.6–70.0)	50.4 (50.1–51.2)
**Family Poverty–Income Ratio** (PIR < 2) (%) **	32.5 (31.0–34.0)	51.0 (42.1–59.8)	32.4 (30.9–33.9)
Ethnicity (%) **			
Non-Hispanic White	67.9 (65.7–70.1)	37.7 (29.5–45.9)	68.1 (65.9–70.3)
Non-Hispanic Black	11.5 (10.3–12.7)	35.5 (28.7–42.3)	11.3 (10.1–12.5)
Hispanic	14.6 (12.8–16.2)	21.5 (15.3–27.7)	14.5 (12.8–16.2)
Other	6.0 (5.5–6.6)	5.3 (2.7–10.0)	6.0 (5.5–6.6)
Education Level (%) **			
Some High School	15.9 (14.8–17.0)	23.5 (17.9–29.2)	15.8 (14.7–17.0)
High School Graduate	22.5 (21.4–23.6)	26.0 (19.1–32.3)	22.5 (21.4–23.6)
Some College or Above	61.6 (59.8–63.3)	50.8 (43.4–58.2)	61.7 (59.9–63.4)
Marital Status (%) **			
Married	56.4 (55.1–57.7)	19.8 (12.0–27.5)	56.7 (55.3–58.0)
Widowed	1.2 (1.0–1.3)	0.4 (0.10–1.6)	1.2 (1.0–1.3)
Divorced	10.0 (9.5–10.5)	6.1 (3.1–11.6)	10.0 (9.5–10.6)
Separated	2.8 (2.6–3.1)	3.6 (2.0–6.4)	2.8 (2.5–3.1)
Never Married	20.2 (19.1–21.4)	55.0 (46.3–63.7)	20.0 (18.8–21.1)
Living with Partner	9.4 (8.6–10.0)	15.2 (9.6–20.7)	9.4 (8.8–10.0)
Depression (%) **	8.0 (7.4–8.6)	15.3 (9.7–20.9)	7.9 (7.3–8.5)

**Note.** * *p* < 0.05 ** *p* < 0.01. Numbers with 95% CI indicate 95% confidence intervals for proportions.

**Table 2:** Risk of depression among adults 20 or older with *C. trachomatis* and C-reactive protein (CRP): NHANES 2005–2010, 20 years and older.

	Total Population OR (95% CI)	*C. trachomatis*− High CRP+ OR (95% CI)	*C. trachomatis*+ High CRP− OR (95% CI)	*C. trachomatis*+ High CRP+ OR (95% CI)
***C. trachomatis*/CRP**	2.77 (1.21–6.34) *	1.25 (1.00–1.75)	2.47 (0.98–6.25)	6.30 (1.18–33.49) *
**Smoking Status**				
Never Smoked (Ref.)	Ref	Ref	Ref	Ref
Former Smoker	1.18 (0.83–1.67)	1.16 (0.82–1.63)	1.13 (0.78–1.62)	1.39 (0.70–2.75)
Current Smoker	2.54 (2.06–3.13) **	2.52 (2.05–3.10) **	2.63 (2.10–3.30) **	1.76 (0.98–3.16)
**Chronic Kidney Disease (CKD)**	1.05 (0.79–1.40)	1.06 (0.79–1.41)	0.93 (0.69–1.26)	1.54 (0.73–3.23)
**Cardiovascular Disease (CVD)**	2.23 (1.55–3.51) **	2.31 (1.53–3.48) **	2.47 (1.64–3.72) **	1.90 (0.87–4.17)
**Diabetes**	1.33 (0.87–2.03)	1.30 (0.85–1.99)	1.34 (0.88–2.04)	1.15 (0.53–2.49)
**BMI**				
Ref BMI ≥ 18.5 and BMI < 25	Ref	Ref	Ref	Ref
BMI ≥ 25 and BMI < 29.9	1.00 (0.79–1.26)	1.00 (0.80–1.26)	0.95 (0.76–1.20) *	1.21 (0.37–3.92)
BMI ≥ 30 and BMI < 34.9	1.32 (0.98–1.77)	1.32 (0.97–1.80)	1.39 (1.01–1.91)	0.61 (0.26–1.46)
BMI ≥ 35.0 and BMI < 39.9	1.27 (0.91–1.78)	1.21 (0.86–1.69)	1.18 (0.79–1.76)	0.89 (0.30–2.66)
BMI ≥ 40.0	1.81 (1.20–2.72) **	1.70 (1.08–2.68)	1.85 (1.12–3.06)	1.11 (0.41–3.00)
**Age (SE)**	1.02 (1.01–1.03) **	1.02 (1.01–1.03) **	1.02 (1.01–1.03) **	1.02 (0.99–1.05)
**Gender (Ref. Female)**	1.90 (1.55–2.33) **	1.86 (1.51–2.29) **	1.90 (1.56–2.32) **	1.73 (0.94–3.21)
**Family Poverty–Income Ratio** (Ref: PIR < 2)	2.55 (2.12–3.06) **	2.60 (2.16–3.11) **	2.53 (2.01–3.18) **	3.00 (1.53–5.89)
**Ethnicity (%)**				
Non-Hispanic White	Ref	Ref	Ref	Ref
Non-Hispanic Black	1.04 (0.84–1.28)	1.01 (0.80–1.27)	1.20 (0.93–1.55)	0.43 (0.22–0.86)
Hispanic	0.96 (0.75–1.24)	0.97 (0.75–1.27)	0.98 (0.74–1.30)	0.74 (0.43–1.29)
Other	0.85 (0.47–1.52)	0.87 (0.49–1.56)	0.90 (0.49–1.65)	0.39 (0.06–2.53)
**Education Level**				
Some College or Above	Ref	Ref	Ref	Ref
Some High School	1.22 (0.98–1.53)	1.21 (0.97–1.53)	1.21 (0.92–1.60)	1.29 (0.69–2.41)
High School Graduate	1.27 (1.02–1.58) **	1.26 (1.01–1.57) **	1.23 (1.00–1.01)	1.46 (0.70–3.04)
**Marital Status**	1.54 (1.28–1.87) **	1.58 (1.29–1.92) **	1.55 (1.26–1.91) **	1.59 (0.91–2.77)

**Note.** * *p* < 0.05 ** *p* < 0.01. OR (95% CI) indicates hazard ratios with 95% confidence intervals for the outcome (i.e., mortality). Ref indicates the reference group among each variable for comparison with other groups.

## Data Availability

Data can be found in the following website: https://wwwn.cdc.gov/nchs/nhanes/default.aspx, accessed on 1 May 2025.
