# Correction: Banerjee et al. Interactive Effect of C-Reactive Protein upon the Relationship Between *Chlamydia trachomatis* and Depression. *Diagnostics* 2025, *15*, 2638

**DOI:** 10.3390/diagnostics16203366

**Published:** 2026-10-16

**Authors:** Kay Banerjee, W. Sumner Davis, Sri Banerjee

**Affiliations:** 1 School of Counseling, Walden University, Minneapolis, MN 55401, USA; karen.banerjee@waldenu.edu; 2 School of Health Sciences and Public Health, Walden University, Minneapolis, MN 55401, USA; dr.sumner.davis@gmail.com

## Text Correction

There was an error in the original publication [1].

(1) Consistent correction of acronyms such as CT to *C. trachomatis* for the sake of correctness (throughout the document)
(2) Consistent correction of years 2005–2010 rather than 2005–2016 or 2005–2018. These were the years that were used. Corrections were made in the abstract and methods section. This does not affect the results.
(3) The following sentence is correct in the abstract “Complex Samples logistic regression models were used to determine if *C. trachomatis* in the previous year was predictive of depression.”; “**Results**: For the presence of depression, the unadjusted Odds Ratio (OR) for *C. trachomatis* to no *C. trachomatis* was OR = 2.9.”
(4) Added a keyword NHANES since this is the dataset that was used.
(5) In the third paragraph of Section 2. Methods, changed “six cycles” to “three cycles”.
(6) In the first paragraph of the results section, the following statement was removed. “As shown in Table 1, individuals with a low sexual frequency experienced an increased (4.0% vs. 2.0%, *p* < 0.001) proportion of mortality compared to individuals with a moderate-to-high sexual frequency.”
(7) The following abbreviations have been expanded with their full names in the main text: NHANES (National Health and Nutrition Examination Survey), the NCHS (National Center for Health Statistics), PHQ-9 (Patient Health Questionnaire-9), AHA (American Heart Association), and CVD (cardiovascular disease). In addition, the later explanations of these abbreviations in the main text have been deleted.
(8) Grammar and language corrections were made to ensure grammatical accuracy and clarity.

## Error in Table

For all of the variables of Table 1, we changed them to percentage (%) as shown below. Age (SE) has been corrected. There was a mistake in Table 2 caption as published. The years that were included were incorrect. The corrected Table 2 caption appears below; some spelling errors in the table were also fixed.

**Table 1:** Characteristics of study participants stratified by *C. trachomatis* status.

Characteristics	Total Population (*n* = 25,938)	*C. trachomatis* (+) (*n* = 271)	*C. trachomatis* (−) (*n* = 25,667)
**C-reactive Protein (%)**	9.2 (8.6–9.7)	12.4 (6.6–18.3)	9.1 (8.6–9.7)
**Smoking Status (%)**			
Never Smoked	53.6 (52.3–54.7)	50.3 (42.6–57.9)	53.6 (52.4–54.8)
Formerly Smoked	20.6 (19.8–21.4)	15.6 (8.4–22.7)	20.6 (19.8–21.5)
Current Smoker	25.8 (24.8–26.9)	34.1 (25.9–42.2)	25.8 (24.7–26.6)
**Chronic Kidney Disease (CKD) (%)**	7.4 (7.0–7.8)	7.8 (4.4–13.6)	7.4 (7.0–7.8)
Cardiovascular Disease (CVD) (%) *	3.7 (3.4–4.1)	1.6 (0.7–3.6)	3.8 (3.4–4.1)
Diabetes (%) **	5.4 (5.0–5.7)	1.7 (0.8–3.6)	5.4 (5.0–5.8)
**BMI (%)**			
BMI ≥ 18.5 and BMI < 25	31.7 (30.7–32.6)	36.9 (29.2–44.6)	31.6 (30.6–32.6)
BMI ≥ 25 and BMI < 29.9	33.1 (32.3–34.0)	28.9 (22.3–35.6)	33.2 (32.3–34.0)
BMI ≥ 30 and BMI < 34.9	19.6 (18.9–20.2)	17.8 (10.0–25.7)	19.6 (18.9–20.20)
BMI ≥ 35.0 and BMI < 39.9	9.0 (8.5–9.4)	8.5 (5.2–13.6)	9.0 (8.5–9.4)
BMI ≥ 40.0	6.7 (6.2–7.1)	7.8 (5.0–11.9)	6.7 (6.2–7.1)
Age (SE) **	39.6 (0.13)	30.2 (30.4)	39.6 (0.13)
**Gender Female (%)**	50.5 (49.9–51.1)	62.1 (54.6–70.0)	50.4 (50.1–51.2)
**Family Poverty–Income Ratio** (PIR < 2) (%) **	32.5 (31.0–34.0)	51.0 (42.1–59.8)	32.4 (30.9–33.9)
Ethnicity (%) **			
Non-Hispanic White	67.9 (65.7–70.1)	37.7 (29.5–45.9)	68.1 (65.9–70.3)
Non-Hispanic Black	11.5 (10.3–12.7)	35.5 (28.7–42.3)	11.3 (10.1–12.5)
Hispanic	14.6 (12.8–16.2)	21.5 (15.3–27.7)	14.5 (12.8–16.2)
Other	6.0 (5.5–6.6)	5.3 (2.7–10.0)	6.0 (5.5–6.6)
Education Level (%) **			
Some High School	15.9 (14.8–17.0)	23.5 (17.9–29.2)	15.8 (14.7–17.0)
High School Graduate	22.5 (21.4–23.6)	26.0 (19.1–32.3)	22.5 (21.4–23.6)
Some College or Above	61.6 (59.8–63.3)	50.8 (43.4–58.2)	61.7 (59.9–63.4)
Marital Status (%) **			
Married	56.4 (55.1–57.7)	19.8 (12.0–27.5)	56.7 (55.3–58.0)
Widowed	1.2 (1.0–1.3)	0.4 (0.10–1.6)	1.2 (1.0–1.3)
Divorced	10.0 (9.5–10.5)	6.1 (3.1–11.6)	10.0 (9.5–10.6)
Separated	2.8 (2.6–3.1)	3.6 (2.0–6.4)	2.8 (2.5–3.1)
Never Married	20.2 (19.1–21.4)	55.0 (46.3–63.7)	20.0 (18.8–21.1)
Living with Partner	9.4 (8.6–10.0)	15.2 (9.6–20.7)	9.4 (8.8–10.0)
Depression (%) **	8.0 (7.4–8.6)	15.3 (9.7–20.9)	7.9 (7.3–8.5)

**Note.** * *p* < 0.05 ** *p* < 0.01. Numbers with 95% CI indicate 95% confidence intervals for proportions.

**Table 2:** Risk of depression among adults 20 or older with *C. trachomatis* and C-reactive protein (CRP): NHANES 2005–2010, 20 years and older.

	Total Population OR (95% CI)	*C. trachomatis*− High CRP+ OR (95% CI)	*C. trachomatis*+ High CRP− OR (95% CI)	*C. trachomatis*+ High CRP+ OR (95% CI)
***C. trachomatis*/CRP**	2.77 (1.21–6.34) *	1.25 (1.00–1.75)	2.47 (0.98–6.25)	6.30 (1.18–33.49) *
**Smoking Status**				
Never Smoked (Ref.)	Ref	Ref	Ref	Ref
Former Smoker	1.18 (0.83–1.67)	1.16 (0.82–1.63)	1.13 (0.78–1.62)	1.39 (0.70–2.75)
Current Smoker	2.54 (2.06–3.13) **	2.52 (2.05–3.10) **	2.63 (2.10–3.30) **	1.76 (0.98–3.16)
**Chronic Kidney Disease (CKD)**	1.05 (0.79–1.40)	1.06 (0.79–1.41)	0.93 (0.69–1.26)	1.54 (0.73–3.23)
**Cardiovascular Disease (CVD)**	2.23 (1.55–3.51) **	2.31 (1.53–3.48) **	2.47 (1.64–3.72) **	1.90 (0.87–4.17)
**Diabetes**	1.33 (0.87–2.03)	1.30 (0.85–1.99)	1.34 (0.88–2.04)	1.15 (0.53–2.49)
**BMI**				
Ref BMI ≥ 18.5 and BMI < 25	Ref	Ref	Ref	Ref
BMI ≥ 25 and BMI < 29.9	1.00 (0.79–1.26)	1.00 (0.80–1.26)	0.95 (0.76–1.20) *	1.21 (0.37–3.92)
BMI ≥ 30 and BMI < 34.9	1.32 (0.98–1.77)	1.32 (0.97–1.80)	1.39 (1.01–1.91)	0.61 (0.26–1.46)
BMI ≥ 35.0 and BMI < 39.9	1.27 (0.91–1.78)	1.21 (0.86–1.69)	1.18 (0.79–1.76)	0.89 (0.30–2.66)
BMI ≥ 40.0	1.81 (1.20–2.72) **	1.70 (1.08–2.68)	1.85 (1.12–3.06)	1.11 (0.41–3.00)
**Age (SE)**	1.02 (1.01–1.03) **	1.02 (1.01–1.03) **	1.02 (1.01–1.03) **	1.02 (0.99–1.05)
**Gender (Ref. Female)**	1.90 (1.55–2.33) **	1.86 (1.51–2.29) **	1.90 (1.56–2.32) **	1.73 (0.94–3.21)
**Family Poverty–Income Ratio** (Ref: PIR < 2)	2.55 (2.12–3.06) **	2.60 (2.16–3.11) **	2.53 (2.01–3.18) **	3.00 (1.53–5.89)
**Ethnicity (%)**				
Non-Hispanic White	Ref	Ref	Ref	Ref
Non-Hispanic Black	1.04 (0.84–1.28)	1.01 (0.80–1.27)	1.20 (0.93–1.55)	0.43 (0.22–0.86)
Hispanic	0.96 (0.75–1.24)	0.97 (0.75–1.27)	0.98 (0.74–1.30)	0.74 (0.43–1.29)
Other	0.85 (0.47–1.52)	0.87 (0.49–1.56)	0.90 (0.49–1.65)	0.39 (0.06–2.53)
**Education Level**				
Some College or Above	Ref	Ref	Ref	Ref
Some High School	1.22 (0.98–1.53)	1.21 (0.97–1.53)	1.21 (0.92–1.60)	1.29 (0.69–2.41)
High School Graduate	1.27 (1.02–1.58) **	1.26 (1.01–1.57) **	1.23 (1.00–1.01)	1.46 (0.70–3.04)
**Marital Status**	1.54 (1.28–1.87) **	1.58 (1.29–1.92) **	1.55 (1.26–1.91) **	1.59 (0.91–2.77)

**Note.** * *p* < 0.05 ** *p* < 0.01. OR (95% CI) indicates hazard ratios with 95% confidence intervals for the outcome (i.e., mortality). Ref indicates the reference group among each variable for comparison with other groups.

A correction has been made throughout the document. The scientific conclusions are unaffected. This correction was approved by the Academic Editor. The original publication has also been updated.
